# Endoscopic far-lateral supracerebellar infratentorial approach for resection of posterior clinoid meningioma: Case report and literature review

**DOI:** 10.3389/fonc.2023.1089002

**Published:** 2023-02-13

**Authors:** Yang Bai, Song Han, Xiaoyu Sun, Xuantong Liu, Xinning Li, Sizhe Feng, Guobiao Liang

**Affiliations:** ^1^ Department of Neurosurgery, General Hospital of Northern Theater Command, Shenyang, Liaoning, China; ^2^ Department of Pathology, General Hospital of Northern Theater Command, Shenyang, Liaoning, China

**Keywords:** posterior clinoid meningioma, supracerebellar infratentorial approach, endoscope, surgical approach, case report

## Abstract

**Introduction:**

The surgery of posterior clinoid meningioma (PCM) remains one of the most formidable challenges for neurosurgeons because of its location at great depth in the cranium and proximity to vital neurovascular structures. Herein, we aim to describe the technique and feasibility of a novel approach, the purely endoscopic far-lateral supracerebellar infratentorial approach (EF-SCITA), for resection of this extremely rare entity.

**Case description:**

A 67-year-old women presented with gradually deteriorating vision in right eye for 6 months. Imaging examinations revealed a right-sided PCM, and the EF-SCITA approach was attempted for tumor resection. Tentorium incision allowed a working corridor toward the PCM in the ambient cistern through the supracerebellar space. During surgery, the infratentorial part of the tumor was found to compress the CN III and posterior cerebral artery medially and encase the CN IV laterally. Following debulking of the infratentorial tumor, the supratentorial part could be exposed and then excised, which had dense adhesions to the ICA and the initial part of the basal vein in front. After total tumor removal, its dural attachment was detected at the right posterior clinoid process and then coagulated under direct vision. The patient on follow-up at 1 month had improvement in visual acuity in right eye, with no restriction of extra-ocular movements.

**Discussion:**

EF-SCITA approach combines advantages of the posterolateral approach and endoscopic technique, allowing access to PCMs with seemingly low risks of postoperative morbidity. It would be a safe and effective alternative for resection of lesions in the retrosellar space.

## Introduction

Posterior clinoid process (PCP) is an uncommon site for the origin of meningiomas, which have only been reported in 18 cases as yet (1–8). Surgical excision of posterior clinoid meningiomas (PCM) presents neurosurgeons with a special challenge since it is deeply nested in the center of cranial base and surrounded by critical neurovascular structures. The complications of surgery for this extremely rare entity are unpredictable, ranging from none to severe disabling neurological deficits. A variety of surgical approaches, including anterolateral, lateral, and posterolateral routes, have been described to access PCM (5). However, the optimal approach to minimize approach-related morbidity and improve the extent of resection still remains to be explored.

Extreme-lateral supracerebellar infratentorial (ELSI) approach remains one of the most versatile approaches in neurosurgery with respect to its ability to address various anatomical regions, especially the posterolateral midbrain and tentorial region (9). In the last few years, the rapid development of endoscopic neurosurgery has brought a great opportunity for the improvement of this approach. Recently, Xie et al. first described the technique of the purely endoscopic far-lateral SCITA (EF-SCITA) for treatment of petroclival region meningiomas (10). We further tested the feasibility of this approach in resection of retroinfundibular craniopharyngioma (CP) in the suprasellar region. Under endoscopy, the retrosellar region and PCP could be clearly exposed through the ambient cistern after tentorium incision (11). This successful experience motivated us to use the same technique for PCM resection.

Herein, we report a case of EF-SCITA in total removal of PCM, with specific emphasis on technical surgical nuances. In the discussion, we summarize breakthroughs and insights on operative approaches for the treatment of PCM, and analyze the operation essentials, and strengths and weaknesses of each passage from the perspective of surgery. In this report, we also review the previous relevant publications within PubMed and abstracts from international conference literature concerning PCM according to the PRISMA guideline. The literature review found a total of 18 cases which was listed in **Table 1**.

**Table 1:** Summary of reported cases of posterior clinoid meningiomas.

Authors	Age (years)	Sex	Symptoms	Surgical approach	Extent of excision	Complications	Pathology
Horiguchi et al. N = 5 (2)	N/D	N/D	N/D	N/D	Simpson G3 (3) Simpson G4 (2)	Permanent hemiparesis (2), permanent CN palsy (4), thalamic dementia (1), perforating artery infarction (3)	N/D
Goto et al. N = 5 (1)	Mean age = 51.6	M (3) F (2)	N/D	Presigmoidal transpetrosal (4) Combined transpetrosal and FTOZ (1)	Total (3) Near total (1) Partial (1)	None	N/D
Ohba et al. N = 1 (8)	60	M	CN III palsy	Transzygomatic	Subtotal	Hemiparesis, hydrocephalus, brainstem infarction	Clear cell
Shukla et al. N = 2 (3)	50	F	HA, diplopia (CN III)	Transzygomatic subtemporal	Total	None	Transitional
	41	F	HA, diplopia (CN III)	Transzygomatic subtemporal	Partial	Hemiparesis, hemianopsia, partial CN III palsy, ACho infarction	Atypical
Sodhi et al. N = 1 (4)	48	F	HA, VD, HH	FTOZ	Near total	Transient ptosis	Meningothelial
Takase et al. N = 2 (5)	54	F	Ophthalamalgia	FT	Near total	None	Meningothelial
	62	F	Akinesia, amnesia, VD, HH	FTOZ	Subtotal	Transient hemiparesis, LSA infarction	Meningothelial
Nanda et al. N = 1 (7)	66	F	Facial pain	Retrosigmoid	Total	Transient hemiparesis	N/D
Young et al. N = 1 (6)	53	F	N/D	Orbitozygomatic transpetrosal	Near total	Mild CN III and IV palsy	N/D
Present case N = 1	67	F	VD	EF-SCITA	Total	None	Meningothelial

ACho, anterior choroidal artery; CN, cranial nerve; EF-SCITA, endoscopic far-lateral supracerebellar infratentorial approach; FT, frontotemporal craniotomy; FTOZ, frontotemporo-orbito-zygomatic craniotomy; HA, headache; HH, homonymous hemianopia; ICA, Internal carotid artery; LSA, lenticulostriate artery; N/D, not described; VD, visual deterioration.

## Case description

A 67-year-old female presented with progressively worsening vision in her right eye for approximately 6 months. On ophthalmological examination, she had mild bilateral peripheral visual field defects, which was severer in right eye. The visual acuity was 0.4 in right eye and 0.8 in left eye. The extraocular muscles were intact and no other neurological deficit was found. Routine laboratory investigations, including endocrinological studies, were normal. No specific past, medical, family and psycho-social history was reported.

**Figure 1:**
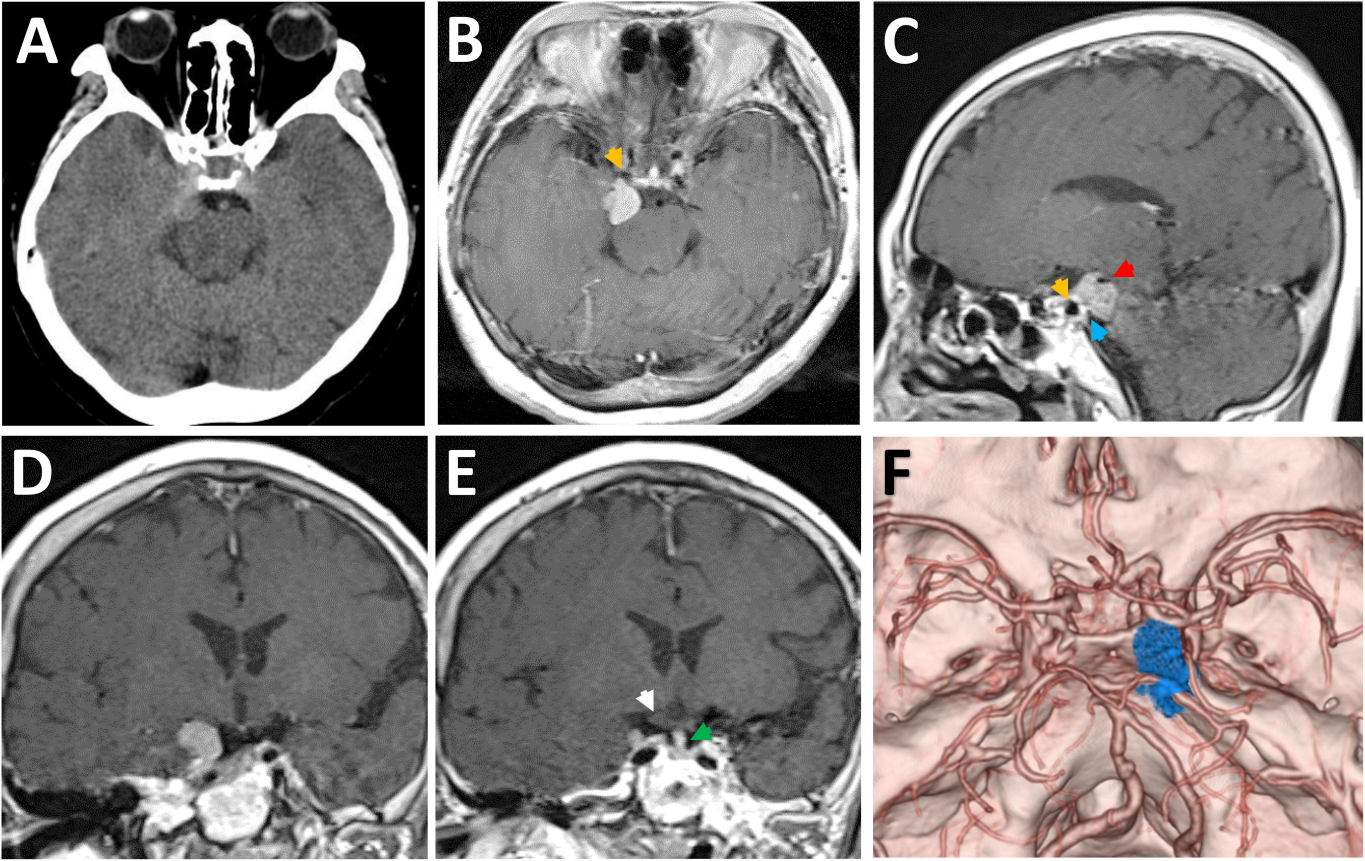
Radiological evaluation of the retrosellar lesion preoperatively. **(A)** Preoperative CT image showing a hyperdense lesion on the right-sided of posterior clinoid process. **(B-E)** Post-contrast (gadolinium-enhanced) axial **(B)**, sagittal **(C)**, and coronal **(D, E)** MRI images showing the thick enhancement of the lesion as well as the relative position to surrounding neural structures. **(F)** Preoperative CT angiography images demonstrating the relative location of tumor to the Willis’ circle and the dorsum sellae. The yellow arrows denote the posterior bend of caveronous carotid artery. The red arrow indicates the posterior cerebral artery. The white arrow denotes the up-elevated right optic chiasma. The green arrow indicates the pituitary stalk. The blue arrow indicates the dural detachment of the tumor on the posterior clinoid process.

Computed tomography (CT) scans revealed a hyperdense lesion on the right side of suprasellar region. The mass grew into the ambient cistern, lying medially close to the medial temporal lobe and displacing the midbrain posteriorly (**Figure 1A**). Magnetic resonance imaging (MRI) examinations showed a homogeneously enhancing tumor centering over the PCP (**Figures 1B–E**). On CT angiography, the tumor was displacing the ICA anteriorly and encasing the right P1 segment of posterior cerebral artery posteriorly (**Figure 1F**). Owing to the mass effect, the right-sided optic chiasma was up-elevated and the pituitary stalk (PS) was slightly displaced to the contralateral side (**Figure 1E**). Based on these clinico-radiological features, PCM was proposed as the most likely diagnosis, and the EF-SCITA approach was attempted for tumor resection (**Video 1**).

**Figure 2:**
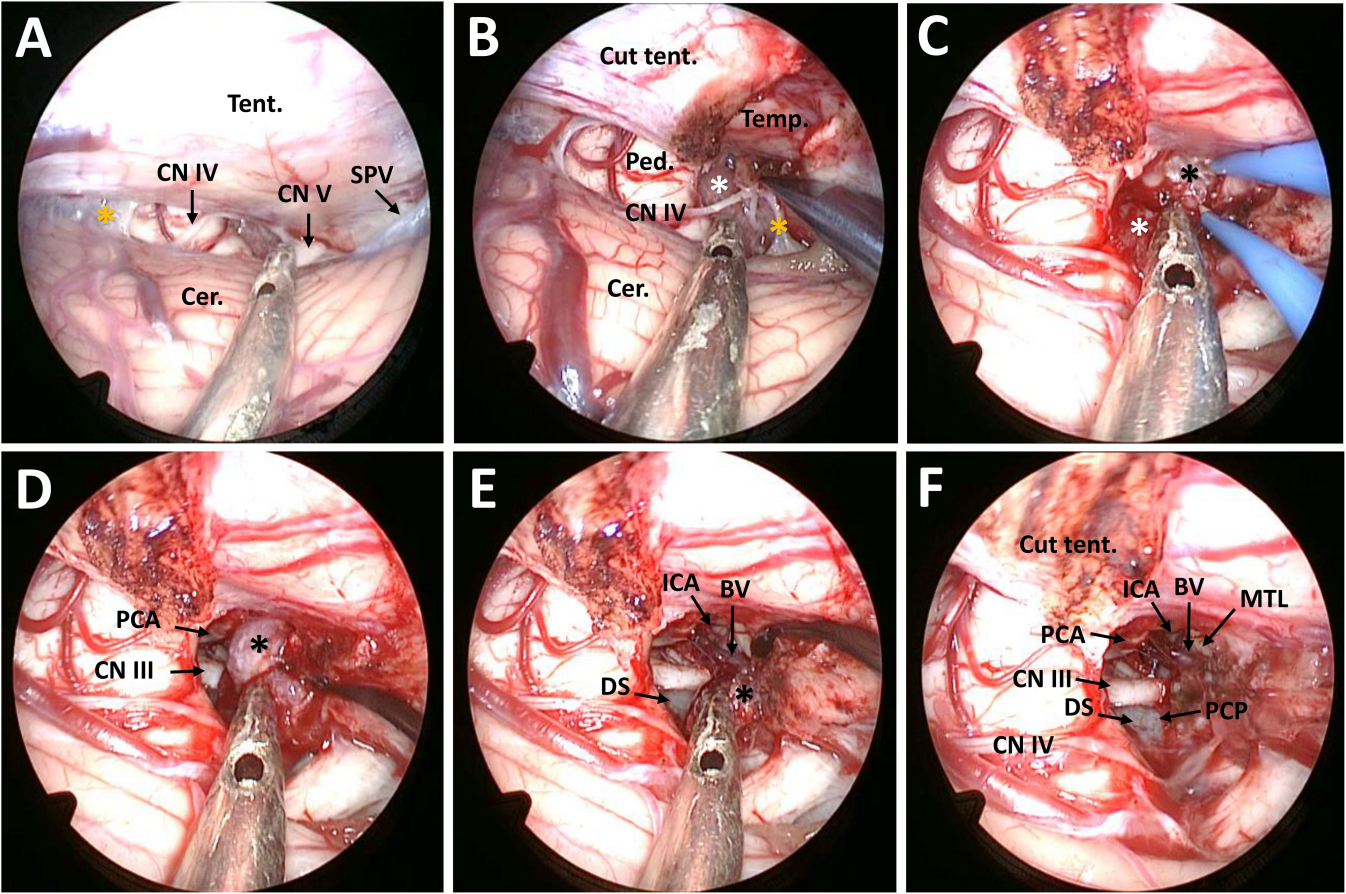
Surgical procedure and nuances of the EF-SCITA approach for resection of posterior clinoid meningioma. **(A)** Endoscopic explorations in the lateral supracerebellar infratentorial space and the exposure of CN IV after opening the arachnoid in the ambient cistern. **(B)** The exposure of the infratentorial part of the tumor in the ambient cistern after incision of the tentorium cerebelli. **(C)** The exposure of the supratentorial tumor after up-elevating the tentorium cerebelli. **(D)** The exposure of the PCA and CN III as well as the supratentorial tumor after debulking of the infratentorial tumor. **(E)** Dissection of the supratentorial tumor from the ICA and the initial part of BV and the exposure of DS during tumor resection. **(F)** The exposure of PCP after total tumor removal. BV, basal vein of Rosenthal (the initial part); Cer., cerebellum; CN III, oculomotor nerve; CN IV, trochlear nerve; CN V, trigeminal nerve; DS, dorsum sellae; ICA, internal carotid artery; MTL, medial temporal lobe; PCA, posterior cerebral artery; PCP, posterior clinoid process; Ped., pedunculus cerebri; PS, pituitary stalk; SPV, superior petrosal vein; Tent., tentorium cerebelli. The black asterisks indicate the supratentorial part of the tumor, the white asterisk denotes the infratentorial part of the tumor, and the yellow asterisks indicate the arachnoid in the ambient cistern.

Surgery was performed in left lateral position after placement of a continuous lumbar CSF drainage system. The head was placed in upper flexion and backward rotation to allow gravity retraction of the cerebellum. As described before, a C-shaped retroauricular incision was performed to obtain adequate exposure of the suboccipital region and the mastoid bone. Then, the entire transverse sinus, the transverse-sigmoid junction, and the proximal part of the sigmoid sinus were revealed through suboccipital craniotomy (11). The surgical position, together with intraoperative drainage, facilitated endoscopic explorations in the lateral superior cerebellar space (**Figure 2A**).

After opening the tentorium cerebelli, the tumor could be identified in the ambient cistern. Anatomically, it was located in front of the midbrain and was divided by the tentorial incisura into the supratentorial and infratentorial parts. The tumor was found to encase and compress the CN IV towards the tentorial incisura (**Figure 2B**). Then, the infratentorial part was excised in a piecemeal fashion. After stepwise tumor reduction, the CN III and the P1 segment located medially to the lesion were identified and the tumor capsule was gently dissected away. In addition, the supratentorial part descended to the surgery field owing to gravity as well as surgical retraction (**Figures 2C, D**). During resection of the supratentorial tumor, we observed that the tumor had dense adhesions to the ICA and the initial part of the basal vein (BV) in front, which were carefully dissected from the tumor (**Figure 2E**). Intraoperatively, the tumor was grayish, soft, and moderately vascular. Total tumor removal was achieved, with all neighboring blood vessels and cranial nerves preserved. Finally, its dural attachment could be detected at the right PCP and adjacent petrous apex, which was coagulated under direct vision (**Figure 2F**).

**Figure 3:**
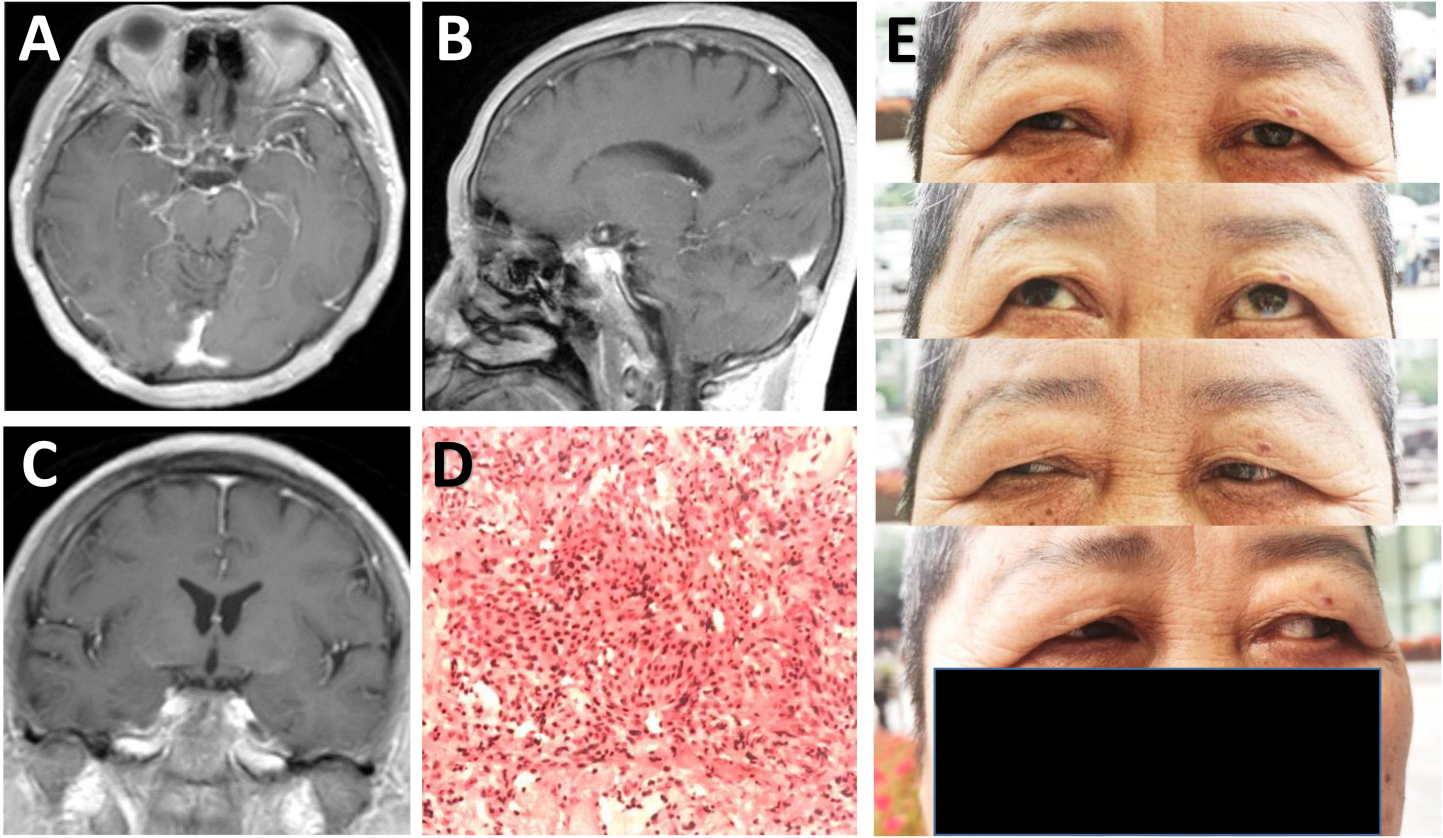
Radiological and pathological evaluation of the lesion postoperatively. **(A-C)** Postoperative post-contrast axial **(A)**, sagittal **(B)**, and coronal **(C)** MRI scans performed one month after surgery showing total removal of the lesion. **(D)** Photomicrograph of hematoxylin-eosin staining showing typical features of meningothelial meningioma. **(E)** Physical examinations at one month follow-up indicating no limitation in eye movements.

The postoperative course was uneventful and the patient was discharged on 7^th^ postoperative day. Postoperative MRI demonstrated complete excision of the lesion (**Figures 3A–C**). Histopathological examinations demonstrated findings consistent with a meningioma of meningothelial type (**Figure 3D**). The patient on follow-up at 1 month had improvement in visual acuity (0.8) in right eye, with no restriction of extra-ocular movements observed (**Figure 3E**).

## Discussion

### Clinical and radiological features of PCM

Due to their rarity and ambiguous terminologies, the true identity of PCMs has only been recognized by Horiguchi et al. in 2008 (2). Later, Sodhi et al. clarified the terminology of lesions in the region of dorsum sellae (DS) and separated PCMs (eccentrically placed meningiomas centering on PCPs) from DS or upper clival meningiomas (centrally located meningiomas between PCPs) (4). On the basis of this definition, there have been only 19 reported cases of PCMs including this study. These patients had a mean age of 54 at the time of diagnosis and most of them were female (71.4%) (**Table 1**).

Anatomically, the PCP is a bony prominence at the superolateral aspect of the DS. Anteromedial to the PCP is the PS. Anterolateral to this process is the posterior edge of the cavernous sinus. The ICA ascends into the posterior aspect of the cavernous sinus where the posterior bend is formed lateral to the PCP. The bifurcation of basilar artery lies posterior to the PCP and DS. The CN III enters the edge of the tentorial dura lateral to the PCP in the oculomotor triangle (12, 13). Thus, meningiomas arising from PCP usually compress the PS anteriorly and the CN III laterally or infero-laterally, and encase the C1-2 segment of the ICA or its branches. The optic chiasm can be even shifted superiorly or antero-superiorly as the tumor expands anteriorly. Therefore, headache, visual disturbance, and diplopia are common presenting symptoms. Huge PCMs also cause facial pain or akinesia if the CN V or ICA perforators is involved (14).

The diagnosis of PCM can be challenging. Radiologically, the “dural tail” sign is seldom detected since the tumor attachment at PCP is very small. Owing to clinical and radiological similarities, some PCMs are preoperatively misinterpreted as DS or upper clival meningiomas, and may be even confused with anterior clinoid meningiomas (ACM) and parasellar meningiomas. The direction of ICA displacement may help in differentiating PCM from ACM, since the ICA is often shifted posteriorly in the case of ACM but anteriorly in the case of PCM (15).

Like most PCMs, the tumor in this case grew upwardly and forwardly beyond the tentorial incisura and up-elevated the right optic chiasma, which could account for progressively visual loss in right eye. However, this case is unique in the tumor site presented unlike those reported in previous studies. Intraoperatively, the tumor was located between CN III and CN IV in the mediolateral direction. Correspondingly, the origin of PCM was found to occupy the junction of the PCP and petrous apex. Thus, the protection of CN IV should also be emphasized during surgery.

### Current surgical strategies for PCM

**Figure 4:**
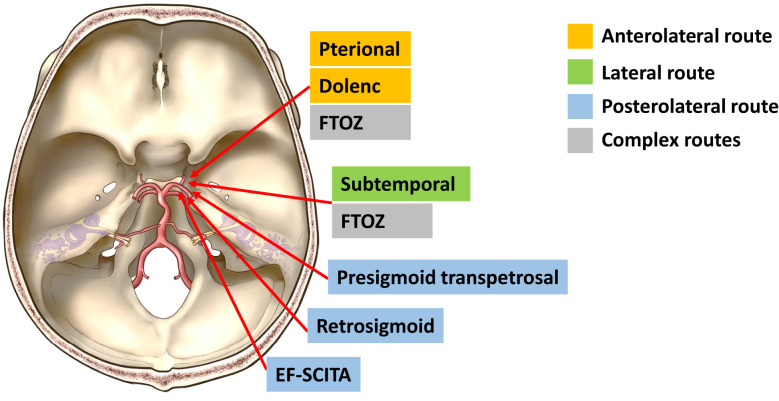
Schematic diagram of surgical approaches for resection of posterior clinoid meningiomas. Current approaches for resection of posterior clinoid meningiomas include anterolateral approaches (the classical pterional approach and the Dolenc approach), the lateral approach (the subtemporal approach), posterolateral approaches (the presigmoid transpetrosal approach, the retrosigmoid approach, and the EF-SCITA approach), and the complex route (the FTOZ approach). FTOZ: frontotemporo-orbito-zygomatic orbitozygomatic approach; EF-SCITA: endoscopic far-lateral supracerebellar infratentorial approach.

The principles of PCM surgery are early devascularization of tumor and careful dissection of tumor from ICA perforators, cranial nerves, PS, and even hypothalamus (1). Several approaches have been implemented to treat this lesion with acceptable risks, and many of them represent variations of the classical pterional, subtemporal, or presigmoid routes with modifications in bone resection. According to surgical directions accessing the PCP, they could be classified into anterolateral, lateral, posterolateral, and complex routes (**Figure 4**).

The transcavernous-transsellar approach was proposed for PCM resection by Dr. Dolenc in 2003. During surgery, the sphenoid wing, ACP, and the lateral wall of the optic canal are excised extradurally. Then, with additional resection of the PCP and DS, the PCM could be devascularized at the attachment and then excised (15). The classical pterional approach allows early identification of ICA perforators and renders direct access to the tumor through the optico-carotid and carotico-oculomotor corridors. Common disadvantages for these anterolateral routes include hindrance of direct approach to tumor by anteriorly shifted ICA and risks to ICA perforators during devascularization and debulking of the tumor through the narrow spaces between them (5, 15). In addition, early detection of CN III is difficult in the transcavernous-transsellar approach (15). Hence, only soft or small PCMs might be treated with these approaches. So far, only one case of reported PCM has been resected *via* fronto-temporal craniotomy with wide splitting of the Sylvian fissure (5).

The inherent drawbacks of anterolateral approaches could be circumvented by lateral or posterolateral approaches. In 2013, Shukla et al. firstly reported two cases of PCM excision *via* transzygomatic subtemporal approach (3). This lateral approach is technically the simplest approach for exposure of tentorial notch and upper clivus. A further incision of the tentorium may be required for a wider exposure. It is a better choice for early devascularization and debulking, and allows for direct access to the PCP between the CN III and posterior communicating artery after resection of small compartment around these structures. Major disadvantages of this approach are temporal retraction and injury to the vein of Labbe, which might be avoided by placing a lumbar drain preoperatively (3).

In 2009, Goto et al. described their experience with five cases of PCM resected *via* presigmoid transpetrosal approach. This posterolateral route guarantees earliest coagulation of feeding arteries of the PCM. Tracing the CN III from the normal proximal side in the interpeduncular cistern to the involved distal side could prevent unexpected injury of the nerve. This approach also enables safe dissection of the antero-superiorly displaced ICA under direct vision and provides direct exposure to the inferior surface of the optic chiasm, PS, and hypothalamus from the posterolateral side. However, burdensome bone drilling with related risks to hearing and facial nerve function as well as cerebrospinal fluid leakage makes it somewhat less desirable surgical choice. Operational complexity also restricts the popularization of this technique (1). In 2017, Nanda et al. reported a case of resection of a large PCM with obvious brainstem compression *via* retrosigmoid approach. Although the most amazing finding was the extent of visualization around the PCP region gained through this approach, the narrow width and great depth of this working corridor pose significant risks of neurovascular injury (7).

Size, consistency, and tumor micromorphology are the most important determinants of the extent of resection and outcome of PCM. Small, soft, and well-delineated tumors can be excised totally with any of the above approaches without significant neurological sequelae, while tumors with large size, hard texture, rich vascularity, and multiple micro-lobules encasing perforators often result in incomplete resection (3). Among the 19 reported PCM cases, total resection was achieved in 9 patients (47.4%) (**Table 1**). It is worth noting that removal of far anterior extension of PCM could not be realized through lateral and posterolateral routes. Under this condition, a basal frontotemporo-orbito-zygomatic orbitozygomatic (FTOZ) approach with wide Sylvian fissure splitting has been recommended. Compared with the classical frontotemporal craniotomy, this approach provides additional anterior temporal or subtemporal route. This surgical trajectory allows direct access to the PCP posterior to the perforating vessels for tumor devascularization and debulking without temporal retraction. Thereafter, the devascularized tumor could be removed through the transsylvian route (4, 5). For extremely huge and complex PCMs, a combined and multi-staged surgical approach could be options of tailor-made surgical strategy. In one reported case, a presigmoid transpetrosal approach was recommended for the first stage combined with an orbitozygomatic transsylvian approach for devascularized residual tumor in the second stage (1).

### EF-SCITA for suprasellar/retrosellar lesions

The ELSI approach was firstly described by Spetzler et al. in 2000 as a distinct variant of the median SCITA approach for accessing the posterolateral mesencephalon (16). Later, it proved its versatility and clinical practicality in treating tumors residing in centrally-located intra-axial structures like the splenium, pulvinar, brainstem, and mesial temporal lobe as well as skull base extra-axial tumors like petroclival meningiomas (9). With the rapid development of neuroendoscopy, the infratentorial space has been recognized as another optimal endoscopic operating area. Earlier anatomic studies have addressed the possibility of endoscope-assisted or purely endoscopic ELSI for accessing the posterior and posterolateral incisural space (17–19). Clinically, endoscope-assisted or purely endoscopic ELSI has been used for removal of supratentorial lesions, such as cavernous malformation in the posteromedial temporal lobe and petroclival meningiomas extending into the middle fossa, with the aid of tentorium incision (10, 20). Based on these studies, we attempted to treat central skull base lesions through this novel approach and have succeed in resection of retroinfundibular CP in a recently reported case (11). Herein, we represented a case of PCM resected *via* the EF-SCITA approach. Despite with the longest working distance, endoscopy extends our eye to see the central skull base and our hands to excise lesions centering over the PCP.

This posterolateral approach necessitates tentorial disconnection to traverse the tentorial incisura and arrive at the retrosellar space, and requires working between neural structures for PCM removal in the ambient cistern. The decompression of infratentorial tumor facilitates the exposure and resection of the part in the supratentorial space. As with transpetrosal approach, this approach allows tracing the natural trajectory of CN III and IV for nerve protection. Based on our prior experience in resection of retroinfundibular CP, this approach also provides direct exposure to the branches of ICA (including the PCA and anterior choroidal artery), optic chiasm and PS from the posterolateral side (11). Thus, this novel approach might be a promising alternative approach for resection of suprasellar or retrosellar lesions.

Compared with the retrosigmoid view, the supracerebellar view supplies the corridor from the medial to cranial nerves and significantly reduces the manipulation of neurovascular complex (21). By exploiting the natural infratentorial space, this corridor is devoid of cortex retraction seen in frontotemporal craniotomy. This approach also avoids other inherent shortcomings of above-mentioned anterolateral, lateral, and posterolateral routes, such as hearing loss, damage to the central skull-base bone, and injury of the Labbe vein. In addition, this technique simplifies craniotomy procedures, with a small incision that does not affects the appearance of patients (11). Another important advantage of this approach is high-definition wide-angle visualization and close-up observation endowed with endoscopy, which further minimize injury especially to various neurovascular structures behind the DS. Concerning the small size of the tumor, only a forward viewing endoscope was utilized in this case. The adoption of angled endoscope will allow visualization of anatomical structures out of the axis of the telescope and minimize the chance of tumor remnants under the condition of large PCMs.

Despite these merits, we are clearly aware that it is not without flaws. Firstly, endoscopy technique has difficulties when handling deep bleeding and necessitates a steep learning curve. Secondly, it is not entirely risk free because it may endanger the brainstem and cerebellum, and has inherent shortcomings of suboccipital craniotomy such as injury to the cerebrovenous system (including the petrosal venous, transverse sinus, and sigmoid sinus). Thirdly, unlike lateral and other posterolateral routes, tumor devascularization could only be achieved after tumor removal owing to the final exposure of PCP. Thus, bleeding control should be stressed especially in resection of massive or easily bleeding tumors. Last but not least, this case represented resection of a small PCM. Considering the surgical corridor, it would be an ideal approach for PCMs with lateral extension to the temporal lobe or posterolateral extension to the petroclival region, but may not be enough for complex PCMs with extreme suprasellar extensions. In this situation, microscopic FTOZ craniotomy may be needed either as an add-on or instead of the EF-SCITA. We believe multi-corridor hybrid surgery *via* combined EF-SCITA and microsurgical middle fossa approach might be an ultimate piece of the surgeon’s armamentarium to improve outcomes in patient with complex DS lesions.

## Conclusion

To the best of our knowledge, this is the first case report describing the EF-SCITA approach in the treatment of meningiomas situated over PCP. Overall, this posterolateral approach is technically simple and appears to be associated with less morbidity, which provides neurosurgeons with a viable alternative to traditional approaches to this kind of extremely rare lesions. However, the efficacy of this approach for larger PCMs awaits further verifications.

## Data availability statement

The raw data supporting the conclusions of this article will be made available by the authors, without undue reservation.

## Ethics statement

Written informed consent was obtained from the individual(s) for the publication of any potentially identifiable images or data included in this article.

## Author contributions

SF, GL, YB and SH have been involved in the operation and management of the patient. SF and GL designed the report. YB and SH reviewed the literature, drafted the article and prepared the figures. XTL and XNL provided important academic inputs during surgery as well as during the revision of this manuscript. All authors contributed in editing of the manuscript and approved its final version. All authors contributed to the article and approved the submitted version.

## Supplementary material

Supplementary Video 1 Video legend This video shows technical specification of endoscopic far-lateral supracerebellar infratentorial approach for resection of posterior clinoid meningioma. Firstly, endoscopic explorations in the lateral supracerebellar space are facilitated by disconnection of the draining vein with bipolar coagulator. Then, the ambient cistern could be exposed by opening ambient cistern arachnoid membranes, and the CN IV and CN V could be revealed in this process. Next, the tumor in the ambient cistern was exposed with the aid of tentorium cerebelli incision, with special attention paid to CN IV protection. The tumor was found to be divided into two parts by the tentorium cerebelli: the infratentorial part and the supratentorial part. Before infratentorial tumor debulking, tumor adhesions to residual ambient cistern arachnoid membranes and the tentorial incisura were separated and removed. The CN IV was found to be encased and compressed towards the tentorial incisura, which was carefully separated from the tumor. In addition, the CN III and the P1 segment of posterior cerebral artery located medially to the lesion were identified, and the tumor capsule was gently dissected away. Along with tumor decompression of the infratentorial part, the supratentorial part descended to the surgery field. During resection of the supratentorial tumor, dense adhesions of this part to the internal carotid artery and the initial part of the basal vein (BV) in front were found, which were carefully dissected from these vital neurovascular structures. Finally, its dural attachment could be detected at the right PCP and adjacent petrous apex after removal of residual tumor, and the dural attachment was coagulated under direct vision.
